# Professional issues in maternal mental health scale (PIMMHS): The development and initial validation of a brief and valid measure

**DOI:** 10.18332/ejm/83276

**Published:** 2018-02-05

**Authors:** Julie Jomeen, Patricia Jarrett, Colin R. Martin

**Affiliations:** 1 Faculty of Health Sciences, University of Hull, Hull, United Kingdom; 2 Independent researcher; 3 Faculty of Society and Health, Buckinghamshire New University, Uxbridge, United Kingdom

**Keywords:** perinatal mental health, psychometrics, midwives, professional issues, education, service development

## Abstract

**INTRODUCTION:**

The life-threatening consequences of perinatal mental health problems (PMHP) are well documented. Midwives are ideally placed to effectively identify women at risk and facilitate early intervention. However, a multitude of factors contribute to failure in recognition and treatment. It would be of value for service providers to be able to identify key professional issues in their own context. The present study sought to develop and evaluate a ‘professional issues in maternal mental health’ scale (PIMMHS), explore its psychometric properties and potential application.

**METHODS:**

A cross-sectional design and instrument evaluation approach was taken to investigate the psychometric properties of the PIMMHS. A total of 266 student midwives from 10 UK institutions completed the PIMMHS via Survey Monkey.

**RESULTS:**

PIMMHS comprises two sub-scales of emotion/communication (PIMMHSEmotion sub-scale) and training (PIMMHS-Training sub-scale). Both PIMMHS subscales demonstrate adequate divergent and convergent validity. Sub-optimal internal consistency was observed for the training sub-scale, however, the PIMMHS-Training had a more impressive effect size in terms of known-groups discriminant validity compared to PIMMHS-Emotion.

**CONCLUSIONS:**

The PIMMHS appears to be a sound psychometric instrument for assessing professional issues that influence the practice of student midwives in PMH. The PIMMHS could support education providers to identify areas for curriculum development, as well as maternity services in proactive assessment of service provision, to identify training and service development opportunities.

## INTRODUCTION

Midwives play a key role in assuring the quality of women’s experiences across the perinatal period, and as such are central to women’s emotional health and wellbeing. Psychological aspects of childbirth and perinatal mental illness (PMI) rose to prominence in the UK following the 2004 Confidential Enquiry in Maternal and Child Health^1^, when for the first time PMI was the largest cause of maternal deaths. This has remained a significant finding in subsequent UK reports^2,3^. However, the consequences of PMHP in terms of maternal, paternal, child and societal life-threatening outcomes is well documented and acknowledged internationally^4^.

In a global context, the assessment and management of perinatal mental health problems (PMHPs) is now an integral part of the midwives’ role, as well as of concern to all practitioners within a maternity context^5^. The perinatal period is a time of high healthcare utilisation^6^, and hence an opportunistic period for the identification of PMHP. Midwives are in a unique and ideal position to effectively identify women at risk and facilitate early intervention^7^. However, a multitude of factors, including reluctance of women to disclose how they are feeling, lack of recognition of the signs of PMHPs by women and healthcare professionals^8^, and a reluctance of professionals to identify affected women because of lack of skills or resources, contribute to failure in recognition and treatment^4,9,10^.

Failure to disclose may well be linked to both stigma and culture. Evidence demonstrates that non-white women and those living in deprived areas are less likely to be asked about mental health^11^, one explanation being that midwives lack the knowledge to manage PMHP across cultures^12,13^. Studies have further identified that a negative attitude to women with PMHPs impacts on professional behaviours, in particular through harmful^14,15^ and generalising stereotypes^16^. Interestingly, stigmatised attitudes can also be expressed in a desire to protect a woman from feeling uncomfortable and/ or being ‘labelled’ and lead to the failure to record mental health history and action a referral into specialist services^14^.

Lack of time in maternity settings and the absence of clearly defined or timely care pathways have also been identified as barriers to the effective prediction and detection of PMHP across a decade of evidence^12,17-20^, as well as a lack of specialist PMH teams and midwives’ knowledge of available options^4^. This is supported by women’s experiences of maternity care when they have mental health problems, which reflects huge inequities in service provision^9^ and lack of knowledge of what services are available^21^.

Noonan et al.^4^, suggest that midwives are constrained in their ability to provide care for women in many ways but that a lack of referral options, educational and organisational supports, as well as busy practice environments, are key areas that influence midwives’ confidence and practice. They state that ‘future research should continue to examine the impact of contextual factors on the provision of PMH care’ (p. 19). To contribute to this agenda, there is a need for service providers to be able to identify what the key professional issues are within their own context. An assessment tool that enables service providers and commissioners to identify those domains of practice where there is a deficit, could facilitate the development of focused service development and training, as well as provide a tool to evaluate any changes made in supporting practitioners to optimise their role in PMH.

The present study sought to develop and evaluate a ‘professional issues in maternal mental health’ scale (PIMMHS) as a composite measure, and to explore the utility of such a measure in terms of psychometric properties and clinical application within both the educational and practice settings. The study will focus on the evaluation of key psychometric attributes, including the factor structure, and validity and reliability of the PIMMHS. Specifically, by asking the following research questions:

1. Can the PIMMHS items be used as a valid and reliable scale?
2. Does the PIMMHS comprise uni-dimensional or multidimensional underlying constructs?
3. Do sub-scales inherent to the PIMMHS demonstrate adequate internal consistency?
4. Do sub-scales inherent to the PIMMHS demonstrate adequate divergent validity?
5. Do sub-scales inherent to the PIMMHS demonstrate adequate convergent validity?
6. Do sub-scales inherent to the PIMMHS demonstrate acceptable known-groups discriminant validity?
7. Do sub-scales inherent PIMMHS sub-scales demonstrate sub-scale discriminability?

## METHODS

### Design and participants

A cross-sectional design was used and an instrument evaluation approach was taken to investigate the psychometric properties of the PIMMHS. Validity and reliability evaluation of the PIMMHS were undertaken using established statistical approaches^22-24^.

### Participants

Ten universities across the UK that provide undergraduate midwifery education took part in the study. Students near the completion of a BSc Midwifery, either undertaking a 3-year or 18-month programme, completed an online questionnaire, delivered via Survey Monkey, focused on issues related to PMH. This group was chosen at this stage of training, due to a perceived exposure to significant theoretical curricula content and practice experience, to answer the questions posed.

The study was presented to students by a midwifery lecturer from their institution at least a week prior to accessing the questionnaire, to provide opportunity for questions or to seek clarification. An email invitation was then sent from the host institution containing a link to a Survey Monkey Questionnaire. One email reminder was sent to students via the administrator working at the students’ University Faculty. The preference was to allow protected time for students to complete the questionnaire, as response rates improved in this circumstance, though students could also complete it independently.

Ethics approval was obtained from the University Ethics Committee of each study site. Consent was embedded at the beginning of the online questionnaire, with 266 questionnaires fully completed.

### Measures

The questionnaire asked for general background information about training in PMH received either during training or in a previous context.

### Professional Issues in Maternal Mental Health Scale ( PIMMHS )

The basis of the development of the PIMMHS was a previous survey questionnaire^19,25^, which focused on a broad range of issues, and aimed to explore confidence, attitudes and knowledge. The survey has been used in a pre-post-test training study, which highlighted that whilst training was useful in positively affecting midwives’ knowledge, skills and attitudes, it was not as impactful in terms of midwives’ judgement of their role in the assessment and management of PMH^25^. We were, therefore, specifically interested in those questions that explored the midwife’s perception of her role in PMH and ability to care for women with PMHP due to environmental aspects, such as time and referral options. Permission to use items from the original scale was obtained from one of the authors (MR-D). For the purposes of this study, the scale was then developed based on the contemporary literature to relate to the now recognised broader set of professional issues in PMH^4^. Questions therefore focused on a number of areas that were potentially considered adaptable, such as the midwives’ and woman’s willingness to discuss PMH, time, location of the assessment, stigma, cultural issues and confidence in the systems, and support available for women with PMH (see Supplementary File 1). The items comprising the PIMMHS were scored on a 0-3 Likert scale with relevant questions reverse scored and higher scores indicating greater agreement with the statement. The adapted scale, as part of a larger project, was piloted with a group of 15 student midwives not included in the main study, who provided feedback on design, clarity, content and format.

### Multi-dimensional Health Locus of Control (MHLC) Scale

Locus of control was determined by adapting the illnessspecific (Form C) version of the Multi-dimensional Health Locus of Control (MHLC) scale^26^. Item content was orientated to the context of PMH. This adaptation assesses four domains of Locus of Control (LC), these being Internal (6-items), Chance (6-items), Doctors (3-items) and Other people (3-items), consistent with the original versions. The MHLC has previously been successfully adapted for the perinatal context^27^. Higher scores indicate greater levels of the particular LC attribute. A 4-point Likert format on a 0-3 rating was used.

### Perinatal Mental Health Awareness-Stress, Anxiety and Depression (PMHA-SAD) sub-scale

The Perinatal Mental Health Awareness (PMHA) scale items were originally developed by an expert panel for use in a study regarding the knowledge and confidence of health visitors in relation to perinatal mental health^28^. The PMHA scale measures: i) knowledge, ii) confidence in identification and, iii) confidence in the management of PMH presentations. The stress, anxiety and depression sub-scale (PMHA-SAD) was further developed for the current study and comprised three questions orientated to the above attributes and scored on a 0-3 Likert scale. Higher scores indicate greater endorsement of the domain.^28^

### Statistical analysis

#### Exploratory factor analysis

Exploratory factor analysis (EFA) was used to determine the factor structure of the PIMMHS scale. The survey and nonscalar use of the items implies unknown factor structure, therefore EFA is appropriate in these circumstances. Maximum-likelihoods (ML) estimation was used for initial factor extraction followed by oblimin rotation of extracted factors since it would be anticipated that in the event of a multidimensional solution emerging, extracted factors would be correlated^29^. The distributional characteristics of items were scrutinised to identify any distributionally non-normal items. These univariate characteristics were compared against the following cut-values^30^ that indicate non-normality; skew values >3 and kurtosis values >10. Multivariate outliers were detected by estimating Mahalanobis distances^31,32^ for each participant. Parallel analysis^33^ was used to estimate the number of underlying factors. The findings from the parallel analysis were corroborated by other statistical indicators, specifically Velicer’s^34^ minimum average partial (MAP) criterion and the Baysian Information Criterion (BIC). A significant item-factor loading was determined by a coefficient level of >0.30, this criterion was chosen to maximise identification of items contributing to the scale and any sub-scales. The comparative fit index (CFI)^35^, the Root Mean Squared Error of Approximation (RMSEA) and the Standardised Root Mean square Residual (SRMR) were used to evaluate model fit using the multiple assessment approach of Bentler & Bonett (1980)^36^. CFI values of 0.95 or greater indicate good model fit^37^, while RMSEA values of less than 0.05 indicate a good fit to the data^38^. SRMR values of 0.05 or less are indicative of good model fit^39^.

#### Divergent validity

Divergent validity was evaluated by correlating PIMMHS scores with the ‘doctors’ sub-scale of the MHLC. No significant relationship was predicted between PIMMHS sub-scales and the ‘doctors’ total sub-scale score.

#### Convergent validity

Convergent validity was evaluated by correlating PIMMHS sub-scale scores with the PMHA-SAD sub-scale. It was predicted that the PIMMHS sub-scale scores would be significantly and positively correlated with the PMHA-SAD sub-scale score.

#### Known-groups discriminant validity

Known-groups discriminant validity was determined by categorising participants on the basis of their dissatisfaction/satisfaction with their training received to date in perinatal mental health. It was predicted that those participants who were categorised as being satisfied with their perinatal mental health training would have significantly higher PIMMHS sub-scale scores in comparison with those categorised as dissatisfied with their perinatal mental health training. Comparisons between groups were evaluated using the between-subject t-test.

#### Internal consistency

The internal consistency of PIMMHS sub-scales was determined using Cronbach’s alpha. A Cronbach’s alpha of 0.70 or greater is considered acceptable^22,40^.

Statistical analysis was conducted using the statistical software package R.

## RESULTS

Two hundred and sixty-six participants took part in the study, the majority being from direct entry programme (N=237) and the remainder from an 18-month short programme for registered nurses (N=29). The smallest number recruited from a single site was N=14 and the largest N=44. The majority of participants (N=191) were aged 30 or younger. All participants were female. Evaluation of Mahalanobis distances revealed the presence of 18 multivariate outliers in the dataset and these participants were consequently excluded from further analysis (final dataset N=248, direct entry N =221 [89%], conversion course N=27[11%]). The means, standard deviations, skew and kurtosis of each PIMMHS item are shown in Table 1 below. Skew and kurtosis characteristics for each item indicate a univariate normal distribution (skew <3, kurtosis <10).

**Table 1:** Distributional characteristics of the Professional Issues Scale (PIMMHS) items

*Item*	*PIMMHS item content*	*Mean*	*SD*	*Skew*	*Kurtosis*
PIMMHS 1	I know exactly who to contact if a woman is experiencing mental health problems	1.87	0.65	-0.23	20
PIMMHS 2	Sometimes I feel reluctant to discuss emotional problems that a woman might be having as I feel uncomfortable discussing these with her	2.03	0.64	-0.21	3.05
PIMMHS 3	Training pays sufficient attention to the cultural dimensions of pregnancy, birth and postnatal care	1.69	0.63	-0.04	2.79
PIMMHS 4	It is easy for me to obtain help for women with mental health problems	1.62	0.63	0.01	2.73
PIMMHS 5	Sometimes I feel reluctant to discuss emotional problems that a woman might be having as I know I am not going to have enough time to deal with them	1.88	0.71	-0.03	2.44
PIMMHS 6	Sometimes I feel reluctant to discuss emotional problems that a woman might be having as I would not know what to do or who to ask for advice	1.90	0.66	-0.06	2.70
PIMMHS 7	There are some emotional issues that women should really not discuss with midwives, are too private and should be discussed with her partner	2.62	0.53	-0.89	2.63
PIMMHS 8	It is difficult to discuss mental health problems with women in the antenatal clinic	1.57	0.76	0.06	2.63
PIMMHS 9	Antenatal clinics are not the best place to discuss a woman’s mental health problems	1.57	0.76	0.02	2.63
PIMMHS 10	Midwives are equipped through training to manage the mental health needs of a woman who has different cultural needs and not originally from the UK	1.23	0.64	0.14	2.99

### Exploratory factor analysis

Parallel analysis suggested that the optimum number of factors was three. However, BIC (-85) suggested two factors and Velicer’s MAP reached a minimum of 0.03, suggesting a one-factor model. To reconcile this inconsistency a three-factor model was specified and item-factor loadings scrutinised following EFA. It was observed that PIMMHS item 7 and PIMMHS item 9 failed to load on any factor (<0.30). PIMMS item 8 ‘Antenatal clinics are not the best place to discuss a woman’s mental health problems’ was observed to load on a factor, in isolation from all other items that loaded (4 items, 3 items) on the two factors. Since a factor cannot comprise a single item^41^, the EFA was re-run eliminating items 7, 8 & 9 and specifying a two-factor solution. The KaiserMeyer-Olkin measure of sampling adequacy (0.81) and the Bartlett test of sphericity (χ^2^=449.15, df=21, p<0.001) indicated the data suitable for EFA. Two factors with eigenvalues greater than 1 (3.02 & 1.21) were found and accounted for 60% of the total variance. Item-factor loadings are summarised in Table 2. Factor 1 comprised items (1, 2, 5 & 6) with an emotional/communicational content and is, hereafter, called the PIMMHS-Emotion sub-scale. Factor 2 comprised items (3, 4 & 10) with content indicative of training requirements and needs, and consequently this is termed the PIMMHS-Training sub-scale. The omnibus goodness-of-it test was nonsignificant (χ^2^=9.70, df=8, p=0.29), indicating excellent model fit. Consistent with this observation CFI=0.99, RMSEA=0.03 (0.01-0.08, 95% CI), RMSR=0.02, dfcorrected RMSR=0.04 suggested excellent model fit. The PIMMS-Emotion and PIMMHS-Training EFA derived sub-scales were found to be significantly and positively correlated (r=0.39, p<0.001).

**Table 2:** Item-factor loadings of the Professional Issues in Maternal Mental Health Scale (PIMMHS) following EFA, significant item-factor loadings are indicated in bold

*Item*	*PIMMHS item content*	*Factor 1*	*Factor 2*
PIMMHS 1	I know exactly who to contact if a woman is experiencing mental health problems	**0.56**	0.11
PIMMHS 2	Sometimes I feel reluctant to discuss emotional problems that a women might be having as I feel uncomfortable discussing these with her	**0.71**	-0.03
PIMMHS 3	Training pays sufficient attention to the cultural dimensions of pregnancy, birth and postnatal care	0.01	**0.52**
PIMMHS 4	It is easy for me to obtain help for women with mental health problems	0.23	**0.48**
PIMMHS 5	Sometimes I feel reluctant to discuss emotional problems that a woman might be having as I know I am not going to have enough time to deal with them	**0.68**	0.04
PIMMHS 6	Sometimes I feel reluctant to discuss emotional problems that a woman might be having as I would not know what to do or who to ask for advice	**0.88**	-0.03
PIMMHS 10	Midwives are equipped through training to manage the mental health needs of a woman who has different cultural needs and not originally from the UK	-0.08	**0.61**

### Divergent validity

No significant correlation was observed between the PIMMHS-Emotion (r=-0.04, p=0.51) and PIMMHS-Training (r=0.09, p=0.16) sub-scales and the MHLC ‘doctors’ subscale score.

### Convergent validity

The PIMMHS-Emotion and the PMHA-SAD measures were observed to be significantly and positively correlated (r=0.41,p<0.001), as were the PIMMHS-Training and PMHASAD measures (r=0.27, p<0.001). The data characteristics of all MHLC sub-scales and the PMHA-SAD sub-scale are summarised in Table 3.

**Table 3:** Multidimensional Health Locus of Control (MHLC) sub-scale scores and Satisfaction item mean score distributional characteristics

*Scale*	*Sub-scale*	*Mean*	*SD*	*Skew*	*Kurtosis*
MHLC	Internal	4.74	.07	0.27	3.34
MHLC	Chance	4.17	.41	0.01	2.49
MHLC	Doctors	4.90	1.10	0.01	3.89
MHLC	Other people	5.79	1.46	-0.30	4.05
PMHA	SAD	5.35	1.27	0.21	3.48

**Figure 1:**
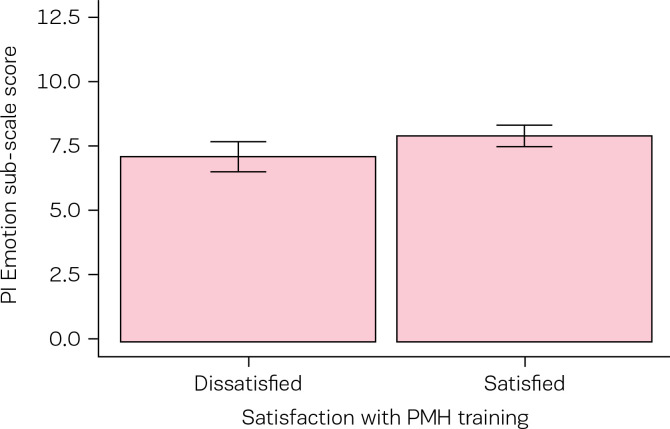
PIMMHS-Emotion sub-scale mean scores as a function of dissatisfaction/satisfaction with perinatal mental health training group classification, error bars represents 95% confidence intervals

**Figure 2:**
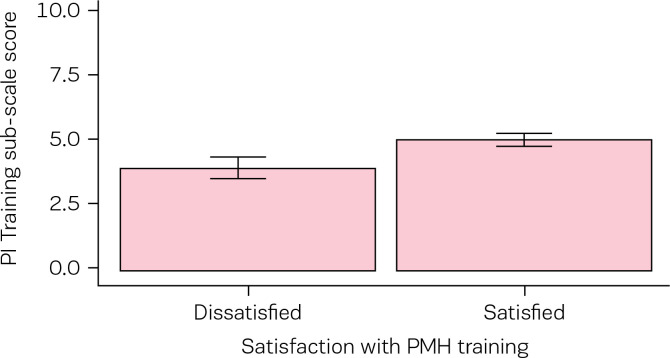
PIMMHS-Training sub-scale mean scores as a function of dissatisfaction/satisfaction with perinatal mental health training group classification, error bars represents 95% confidence intervals

Dichotomous categorisation of participants based on their dissatisfaction/satisfaction with perinatal mental training revealed that the majority of participants (N=192;77%) were satisfied with their training in this area. Independent t-tests revealed statistically significant differences between groups on the PIMMHS-Emotion sub-scale in the direction predicted (Table 4, Figure 1). Similarly, statistically significant differences between groups were observed on the PIMMHS-Training sub-scale in the direction predicted (Table 4, Figure 2).

**Table 4:** Mean PIMMHS Emotion and Training sub-scale scores as a function of group status classified by their satisfaction (Dissatisfied/Satisfied) with training in perinatal mental health (N=248), standard deviations are in parentheses

*PIMHHS sub-scale*	*Dissatisfied (N=56)*	*Satisfied (N=192)*	*t*	*df*	*p*	*Cohen’s d*	*95% CI*	*Effect size*
PIMMHS-Emotion	7.07 (2.26)	7.86 (2.05)	2.47	246	0.01	0.38	0.07 - 0.67	small
PIMMHS-Training	3.91 (1.33)	4.73 (1.36)	4.01	246	<0.001	0.61	0.30 - 0.91	med

### Internal consistency

Cronbach’s alpha of the PIMMHS-Emotion sub-scale was 0.91 (0.89-0.92) and the PIMMHS-Training sub-scale, 0.57 (0.48-0.66). Parentheses indicate 95% confidence intervals (CI).

## DISCUSSION

The current investigation sought to determine if the PIMMHS items could be used as a scaled and psychometrically robust measure for assessing professional issues of salience to PMH in the educational and applied setting. It was noted that descriptive review of the individual item distributional characteristics suggested the suitability of the items to be explored, in terms of a robust psychometric appraisal of measurement qualities, using a statistical approach underpinned by parametric assumptions of data normality.

Prior to conducting the EFA, a range of statistical approaches were undertaken with a view to determining consensus on the number of underlying factors likely to comprise the scale. Parallel analysis represents a contemporary and statistically robust approach, however, the number of factors suggested by this method (three) was inconsistent with both BIC (two) and Velicer’s MAP (one). This inconsistency was addressed by a careful and detailed review of item-factor loadings based on a three-factor EFA. It was noted that a key issue that likely promoted the inconsistency observed was the single-item item-factor loading of item 8 ‘It is difficult to discuss mental health problems with women in the antenatal clinic’. A factor cannot be reasonably defined by a single-item therefore the simplest approach to adopt was to rerun the analysis as a two-factor solution with item 8 and non-factor loading items 7 and 9 removed. It was noteworthy that the consequent two-factor solution yielded an excellent fit to the data according to all fit indices therefore offering convincing evidence for a twofactor solution and thus, extrapolating from this, that the PIMMHS comprises two sub-scales. Evaluation of the items in terms of their respective loading onto factors suggests sub-scales of emotion/communication (PIMMHS-Emotion sub-scale) and training (PIMMHS-Training sub-scale), (Supplementary File 2). It is interesting here to speculate on the content of the questions that were removed following the factor analysis. Question 7, which relates to emotional issues not being ones that women should really discuss with midwives, may be related to a now relatively consistent acceptance by midwives in their role in PMH^4,18^, making this question somewhat redundant. Questions 8 and 9 on the antenatal clinic environment, may not be dissimilar being underpinned by the general acceptance and the growing acknowledgement of the range of PMHP^42,43^ and value of identifying women with potential PMHP in the antenatal period^5,44^, albeit problems often still remain with effective and consistent identification^8^, as well as good record keeping^4^.

The potential value and utility of the PIMMHS-Emotion and PIMMHS-Training sub-scales may be inferred by the findings of the validity and reliability observations. It was noted that not only did both PIMMHS sub-scales demonstrate adequate divergent and convergent validity but also the known-groups discriminant validity evaluation demonstrated the sensitivity of the scales to student’s satisfaction/dissatisfaction with their training in perinatal mental health. This is fundamentally important as it offers an important insight into the potential impact of training on later practice, assuming that measures map onto clinical engagement behaviour and also on the content of such training. Given that the PIMMHS-Emotion sub-scale relates to emotional burden, it is perhaps surprising, given that the training itself is mental health related, that the content of training generally does not address or reflect upon the potential emotional burden to the student midwife herself during the training process. Whilst this could be considered a speculative claim, previous literature has identified the gaps in education and training in addressing the emotional impact that supporting women can have on midwives and calls for the inclusion in training and education of content such as, emotion work skills including professional boundary setting, stress management and healthy coping strategies^45^. Omission of this content may potentially promote cognitive dissonance during training, which leads to dissatisfaction with the training itself and ultimately impacts on the quality of clinical care in the event of post-qualification encountering of women with PMHP. Role modelling and clinical supervision, incorporated in training, are identified as mechanisms to enhance midwives’ self-efficacy to provide emotional care to women^4^. Hence, professional development and training in PMH that begins in the undergraduate midwifery education programmes must be continued through CPD and at post-registration/graduate levels, as fundamental to the development of confidence and enhancement of PMH care provision^4,15^. A sizable minority of participants were dissatisfied with their perinatal mental health training and the robustness of the measure itself (PIMMHS) appears sensitive to this effect in terms of scores on both PIMMHS sub-scales; this observation would suggest a review of curricula content of PMH education and training to ensure optimum engagement and enhanced benefit for both midwives and women.

One caveat to the otherwise impressive psychometric observations observed thus far must be the sub-optimal internal consistency observed for the PIMMHS-Training sub-scale, in stark contrast to the excellent internal consistency observed for the PIMMHS-Emotion sub-scale. The significance of sub-optimal internal consistency for the PIMMHS-Training sub-scale is difficult to determine at this early stage, however it should be recognised that internal consistency is influenced by scale length, thus three items represents a realistic minimum for a sub-scale and it may be anticipated that alpha would be modest at best with so few items^41^. Contrasting this finding, is though, the observation that in terms of known-groups discriminant validity, the comparison between groups revealed the PIMMHS-Training sub-scale to have the more impressive effect size compared to the PIMMHS-Emotion sub-scale. Further enquiry would be extremely valuable in determining whether the PIMMHSTraining sub-scale may need revision, potentially with additional items, to improve internal consistency; or whether the functionality of this sub-scale ‘as is’ is of a degree of robustness, given the other observed psychometric parameters, to be both suitable and appropriate for use in the current version.

The study had a small number of limitations that are readily addressed through future development work on the measure. The survey design approach used for data collection, while valid for initial development of the tool, does not allow the opportunity to evaluate test-retest reliability, a valuable additional index of psychometric integrity. Further work should look to evaluate the test-retest reliability of the PIMMHS sub-scales using a 12-week pre-post repeatedmeasures design consistent with the recommendations of Kline^22^. Value would also be gained by evaluating the sensitivity of the PIMMHS sub-scales to intervention, to fully determine the performance attribute of the measure.

## CONCLUSIONS

The PIMMHS appears to provide a sound psychometric instrument for assessing those professional issues that influence practice of student midwives in PMH. It offers the opportunity to robustly assess modifiable factors in practice that are essential to the provision of high quality care provision for PMH. Evidence highlights that a multi-faceted approach to PMH care incorporating education programmes and other support systems, such as clinical supervision and improved access to specialist guidance, are essential. The PIMMHS could support maternity services in proactive assessment of service provision, which could then underpin the identification of training need, as well as service development opportunities. In addition, findings could be used in an educational context to develop curriculum related to PMH. PIMMHS could then be used ‘post intervention’ to engage in robust evaluation of any curricula development, training package delivery or service change ultimately supporting optimal PMH provision for women within maternity services and promoting improved outcomes for mother, child and wider family. Further testing of the PIMMHS in qualified midwives and other groups would be of value.

**Supplementary Materials:** The following can be made available online at http://www.europeanjournalofmidwifery. eu/Issue-February-2018,3457. Figure S1: **Pre psychometric testing version of the PIMMHS;** Figure S2: **Postpsychometric testing of the PIMMHS.**
