# Supplementary material for: Professional issues in maternal mental health scale (PIMMHS): The development and initial validation of a brief and valid measure
Source: Eur J Midwifery. 2018 Feb 5;2:2. doi: 10.18332/ejm/83276 (PMC7846036; doi:10.18332/ejm/83276)
Supplement: Supplementary file 1 [file EJM-2-2-s1.pdf]

Supplementary File 1: Pre psychometric testing version of the PIMMHS

Professional Issues in Perinatal Mental Health		*Strongly Agree*	*Agree*	*Neither Agree nor Disagree*	*Disagree*	*Strongly Disagree*
**Q1.**	I know exactly who to contact if a woman is experiencing mental health problems	☐	☐	☐	☐	☐
**Q2.**	Sometimes I feel reluctant to discuss emotional problems that a women might be having as I feel uncomfortable discussing these with her	☐	☐	☐	☐	☐
**Q3.**	Training pays sufficient attention to the culture dimensions of pregnancy, birth and postnatal care	☐	☐	☐	☐	☐
**Q4.**	It is easy for me to obtain help for women with mental health problems	☐	☐	☐	☐	☐
**Q5.**	Sometimes I feel reluctant to discuss emotional problems that a woman might be having as I know I am not going to have enough time to deal with them	☐	☐	☐	☐	☐
**Q6.**	Sometimes I feel reluctant to discuss emotional problems that a woman might be having because I would not know what to do or who to ask for advice	☐	☐	☐	☐	☐
**Q7.**	There are some emotional issues that women should really not discuss with midwives, are too private and should be discussed with her partner	☐	☐	☐	☐	☐
**Q8.**	It is difficult to discuss mental health problems with women in the antenatal clinic	☐	☐	☐	☐	☐
**Q9.**	Antenatal clinics are not the best place to discuss a woman's mental health problems	☐	☐	☐	☐	☐
**Q10.**	Sometimes I feel reluctant to discuss emotional problems that a woman may be experiencing as I don't feel adequately trained to deal with these issues	☐	☐	☐	☐	☐

Supplementary File 2: Post psychometric testing version of the PIMMHS

Professional Issues in Perinatal Mental Health		*Strongly Agree*	*Agree*	*Neither Agree nor Disagree*	*Disagree*	*Strongly Disagree*
	**PIMMHS-Emotion**					
**Q1.**	I know exactly who to contact if a woman is experiencing mental health problems	☐	☐	☐	☐	☐
**Q2.**	Sometimes I feel reluctant to discuss emotional problems that a women might be having as I feel uncomfortable discussing these with her	☐	☐	☐	☐	☐
**Q3.**	Sometimes I feel reluctant to discuss emotional problems that a woman might be having as I know I am not going to have enough time to deal with them	☐	☐	☐	☐	☐
**Q4.**	Sometimes I feel reluctant to discuss emotional problems that a woman might be having because I would not know what to do or who to ask for advice	☐	☐	☐	☐	☐
	**PIMMHS-Training**					
**Q5.**	Sometimes I feel reluctant to discuss emotional problems that a woman may be experiencing as I don't feel adequately trained to deal with these issues	☐	☐	☐	☐	☐
**Q6.**	Training pays sufficient attention to the culture dimensions of pregnancy, birth and postnatal care	☐	☐	☐	☐	☐
**Q7.**	It is easy for me to obtain help for women with mental health problems	☐	☐	☐	☐	☐
